# Diel Niche of Sympatric Small Mammals Revealed by Year‐Round Camera Trapping

**DOI:** 10.1002/ece3.71590

**Published:** 2025-06-12

**Authors:** Lars K. Lindsø, Inger Maren Rivrud, Emilie A. König, Anders Herland, Atle Mysterud

**Affiliations:** ^1^ Centre for Ecological and Evolutionary Synthesis (CEES), Department of Biosciences University of Oslo Oslo Norway; ^2^ Norwegian Institute for Nature Research (NINA) Oslo Norway; ^3^ Norwegian Institute for Nature Research (NINA) Trondheim Norway

**Keywords:** camera traps, diel niche, non‐invasive sampling, small mammals

## Abstract

At northern latitudes, small mammals are of key importance for ecosystem dynamics. Yet, few have monitored the small mammal community to accurately investigate activity of sympatric small mammals at finer temporal resolutions, and consequently their temporal niche partitioning that may provide novel insights into how they interact with their environment and each other. Here, we deployed 12 tunnel‐based camera traps to continuously monitor a forest community of small mammals in South‐East Norway (2018–2020). We statistically determined the diel period selection (cathemeral, nocturnal, diurnal, crepuscular) in the wood mouse ( *Apodemus sylvaticus* ), bank vole ( *Myodes glareolus* ), common shrew ( *Sorex araneus* ) and pygmy shrew ( *S. minutus* ). We analyzed how temperature and snow cover influenced small mammal occurrence in winter and whether camera trap observations correlated with numbers from ordinary trapping in spring and fall. In both winter and summer, wood mice were nocturnal, while common shrews were cathemeral. Diel period selection in bank voles and pygmy shrews varied seasonally, and both were cathemeral in summer and nocturnal in winter. The occurrence of both rodents and shrews developed from a low year in 2018, to an increase in 2019, and to a peak year in 2020, albeit at lower amplitudes for the pygmy shrew. Small mammal occurrence in winter was positively influenced by temperature, but the relationship was dependent on snow cover and was only significant at low snow levels. Camera trap occurrence correlated moderately with spatially and temporally coinciding ordinary trapping indices in spring and fall. Thus, tunnel‐based camera traps captured a new level of detail in species synchrony of common small mammals in northern Europe at annual, seasonal, and diel scales, yielding a better understanding of their ecology.

## Graphical abstract

Small mammals are of key importance for many ecosystems, but few have monitored their communities non‐invasively and at high temporal resolutions. Here, we deployed 12 tunnel‐based camera traps to continuously monitor a forest community of rodents and shrews in South‐East Norway (2018–2020) and statistically determined their season‐specific diel niche. We found differences in both diel phenotype (cathemeral, nocturnal) and levels of nocturnality between species and between seasons, highlighting how continuous camera trapping and a novel statistical tool can yield a better understanding of small mammal ecology.

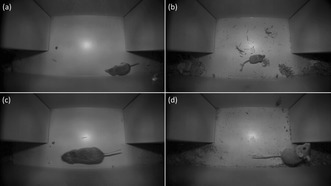

## 1 Introduction

Traditionally, studies of wildlife ecology have focused on spatial habitat use, emphasizing where species occur and how different species partition physical space to reduce competition and maximize access to resources. However, temporal habitat use is increasingly recognized as an equally important aspect of ecology (Anderson and Wiens 2017; Rivera et al. 2022; Cox and Gaston 2024). Indeed, the daily activity pattern of a species, that is, the diel period selection, represents a fundamental part of its ecological niche (Halle and Stenseth 2012; Bennie et al. 2014). The diel niche is often defined by whether its activity is focused on specific diel periods (Anderson and Wiens 2017; Cox and Gaston 2024). Different species may respond differently to the cost or benefit of being active during a given diel period due to interspecific avoidance and predation (Greenwood 1978; Monterroso et al. 2013; Cunningham et al. 2019; Gaynor et al. 2019), differences in diet, metabolism, and foraging behavior (Refinetti et al. 2016), and in response to human disturbance (Gaynor et al. 2018; Hoffmann et al. 2018). Consequently, some species may show adaptability in diel period selection in response to shifting environmental contexts (Rivera et al. 2022; Cox and Gaston 2024). However, studies on diel period selection in wildlife commonly lack explicit quantitative definitions for diel niches and shifts in diel period selection. This imposes problems for evaluating hypotheses empirically, cross‐study comparisons, and assigning diel niches with measures of uncertainty (Gerber et al. 2024).

Small mammals play a key role in ecosystem structure and function at northern latitudes (Ims and Fuglei 2005; Krebs 2010; Boonstra et al. 2016), but are cryptic and resource demanding to study. Moreover, shifting seasons drive marked environmental changes related to temperature and photoperiod (Ergon et al. 2004; Helm et al. 2013), which may influence diel period in small mammals selection due to changes in energy balance and expenditure (van der Vinne et al. 2014; Guiden and Orrock 2020), in response to shifting light levels through the day (Halle 2006), and predation risk (Penczykowski et al. 2017). Historically, most small mammal monitoring programs are based on conventional live or lethal trapping, whereby animal counts are used as indices of abundance (Cornulier et al. 2013; Korpela et al. 2013; Ehrich et al. 2020; Sørensen et al. 2022). However, these methods are beset with ethical issues (Powell and Proulx 2003) and may impose questionable sampling‐related assumptions (Hanski et al. 1994). Camera trapping has been widely applied in wildlife studies on larger animals in the last decades as a non‐invasive alternative (Burton et al. 2015). Recent developments in camera trapping have also included camera traps specifically adapted to study small mammals under the snow. Tunnel‐based camera traps can be deployed year‐round in seasonal environments, enabling continuous monitoring of animal occurrence (Soininen et al. 2015). Importantly, camera traps have the potential to yield information on population parameters at higher temporal resolutions and at a lower resource cost compared to other conventional methods and can be adapted at both local and larger spatial scales (Kleiven et al. 2023).

Here, we deployed 12 tunnel‐based camera traps continuously between 2018 and 2020 to monitor year‐round occurrence of small mammals in South‐East Norway. We here quantify the respective season‐specific diel period selection probabilities in different species of both rodents and insectivores, using a novel statistical framework (Gerber et al. 2024). We predicted that (I) diel period selection in *Apodemus* mice was consistent with a nocturnal phenotype and that *Myodes* voles and *Sorex* shrews were more evenly distributed through the day, that is, a cathemeral phenotype. Furthermore, we predicted that (II) seasonal shifts in diel period selection would favor increased cathemerality, that is, more even diel activity, in winter due to increased energy requirements for thermogenesis in cold temperatures and reduced predation risk under snow cover. For annual and seasonal occurrence, we predicted that (III) patterns were similar across all species but at higher amplitudes in rodents compared to shrews due to higher annual reproduction in rodents. Furthermore, we also tested whether winter occurrence of small mammals was influenced by climatic factors linked to temperature and snow cover. We predicted that (IV) temperature and snow cover positively influenced winter occurrence due to increased survival and reduced predation risk. Lastly, we tested whether camera trap occurrence counts and capture indices from conventional live and lethal trapping were correlated using spatially and temporally overlapping surveys.

## 2 Materials and Methods

### 2.1 Study Area

**FIGURE 1:**
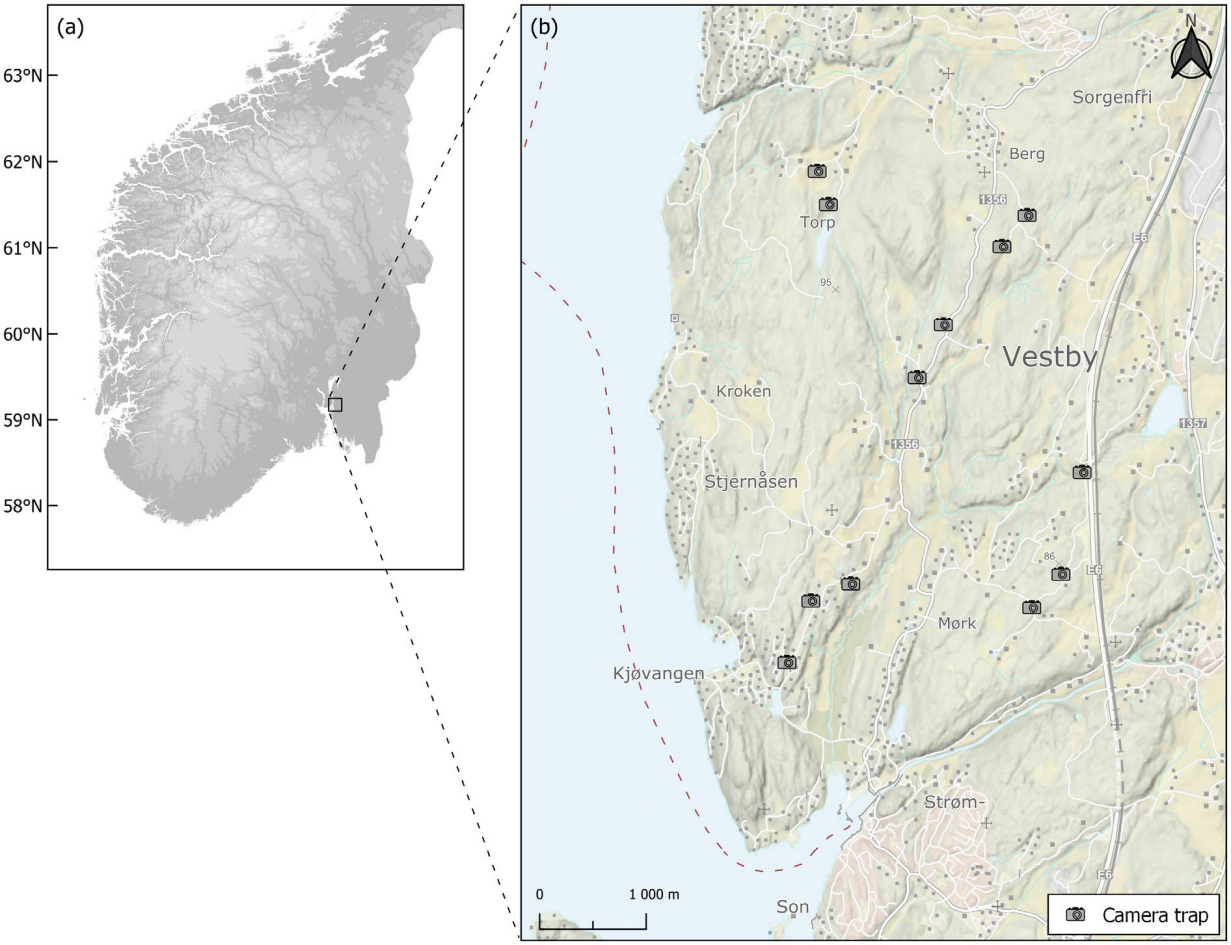
Map of camera trap locations. The map shows (a) the main location of the study area in Southern Norway and (b) the spatial distribution of 12 camera trap sites.

The study was conducted in Vestby, Akershus, in South‐East Norway (Figure 1). The study area encompasses a habitat mosaic of smaller settlements, grain farmland, and managed forests. The area falls within the boreonemoral zone (Abrahamsen et al. 1977) with an average annual temperature of 7.5°C and an average total precipitation of 868 mm (Norwegian meteorological station no. 17380 Rygge‐Huggenes, 2020–2024, met.no). The predominant tree species in forested areas include Scots pine ( *Pinus sylvestris* ) and Norway spruce ( *Picea abies* ), birch (*Betula* spp.), oak (*Quercus* spp.), and elm (*Ulmus* spp.). The forest undergrowth predominantly consists of bilberry ( *Vaccinium myrtillus* ), heather ( *Calluna vulgaris* ), peat moss (*Sphagnum* spp.), and various forbs and graminoids. Known small mammals in the area include bank vole ( *Myodes glareolus* ), field vole ( *Microtus agrestis* ), wood mouse ( *Apodemus sylvaticus* ), common shrew ( *Sorex araneus* ), and pygmy shrew ( *Sorex minutus* ) (Lindsø et al. 2023). In a previous study, spatial variation in occupancy of these species was low in the study area and was not explained by available habitat characteristics, including vegetation composition and structure (Munkelien 2018). Medium‐ to large‐sized mammals in the area include red squirrel ( *Sciurus vulgaris* ), red fox ( *Vulpes vulpes* ), badger ( *Meles meles* ), roe deer ( *Capreolus capreolus* ) and moose ( *Alces alces* ) (Mysterud et al. 2021).

### 2.2 Camera Trapping and Ordinary Trapping

**FIGURE 2:**
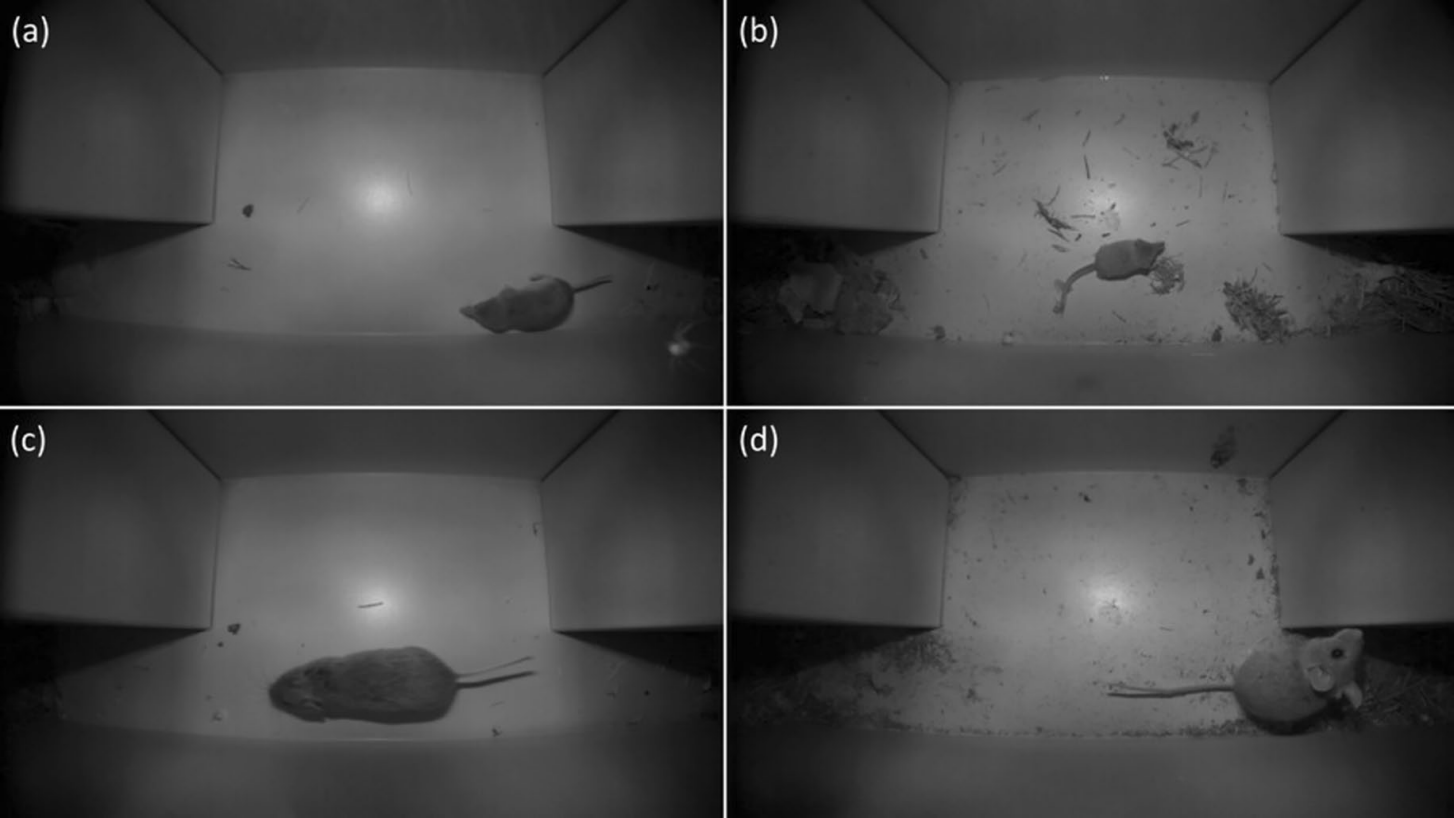
Examples of camera trap images of different small mammal species; (a) common shrew ( *Sorex araneus* ), (b) pygmy shrew ( *Sorex minutus* ), (c) bank vole ( *Myodes glareolus* ), and (d) wood mouse ( *Apodemus sylvaticus* ).

We deployed camera traps all year‐round between July 2018 and December 2020 at 12 forest sites (Figure 1). We used a tunnel camera trap designed for year‐round use (Soininen et al. 2015). The trap consisted of an aluminum tunnel with a movement‐triggered camera (Reconyx PM750 Professional) attached to the roof. The camera records passing animals at a fixed distance, enabling species identification (Figure 2). The camera was set to record three pictures in quick succession upon being motion‐triggered. The device was placed along typical small mammal runways identified by clues in the field, for example, between rocks or mounds. All cameras were inspected for maintenance twice every year to replace the camera's battery and memory card.

We captured small mammals for three consecutive days in both late May and late August (2018–2020), as detailed in previous studies (Mysterud et al. 2019; Lindsø et al. 2023). Within 20 m of each camera trap site, we had four trap stations according to the small quadrate method (Myllymäki et al. 1971). Each trap station consisted of either one cage trap (Ugglan Special No. 3, Grahnab AB) or three common snap traps (Norwegian mouse trap Rapp 1) to avoid trap saturation. A carrot was placed inside cage traps to sustain trapped animals, and a raisin was placed on the snap trap trigger. All traps were baited with oats and checked daily for three consecutive days. All animals found alive were culled by cervical dislocation to allow for examination of their parasites and pathogens, presented in a different study (Lindsø et al. 2023). All captured animals were stored in plastic bags and later determined to species by a small mammal expert (Jeroen van der Koijj).

The minimum distance between trap sites was 500 m to avoid recording the same individual small mammal in more than one trap and to ensure independent occupancy rates between the traps (Mölle et al. 2022) and to avoid local depletion of populations with lethal trapping (Mysterud et al. 2019).

### 2.3 Camera Trap Data

We manually identified the species and number of individuals present in each picture. Pictures of humans taken during camera trap deployment or maintenance were discarded. All non‐empty pictures were assigned an event number. One event was defined as all pictures of the presumed same individual animal within exactly 60 s of the next animal observation (picture). If an animal with clearly different characteristics, that is, typically size, triggered the camera within 60 s of the previous animal, or if sequences of pictures within 60 s were followed by pictures with two individuals, a new event number was assigned. We visualized annual and season‐specific time‐of‐day patterns of species occurrence using locally weighted smoothing (loess) of camera trap events.

### 2.4 Statistical Analyses

**FIGURE 3:**
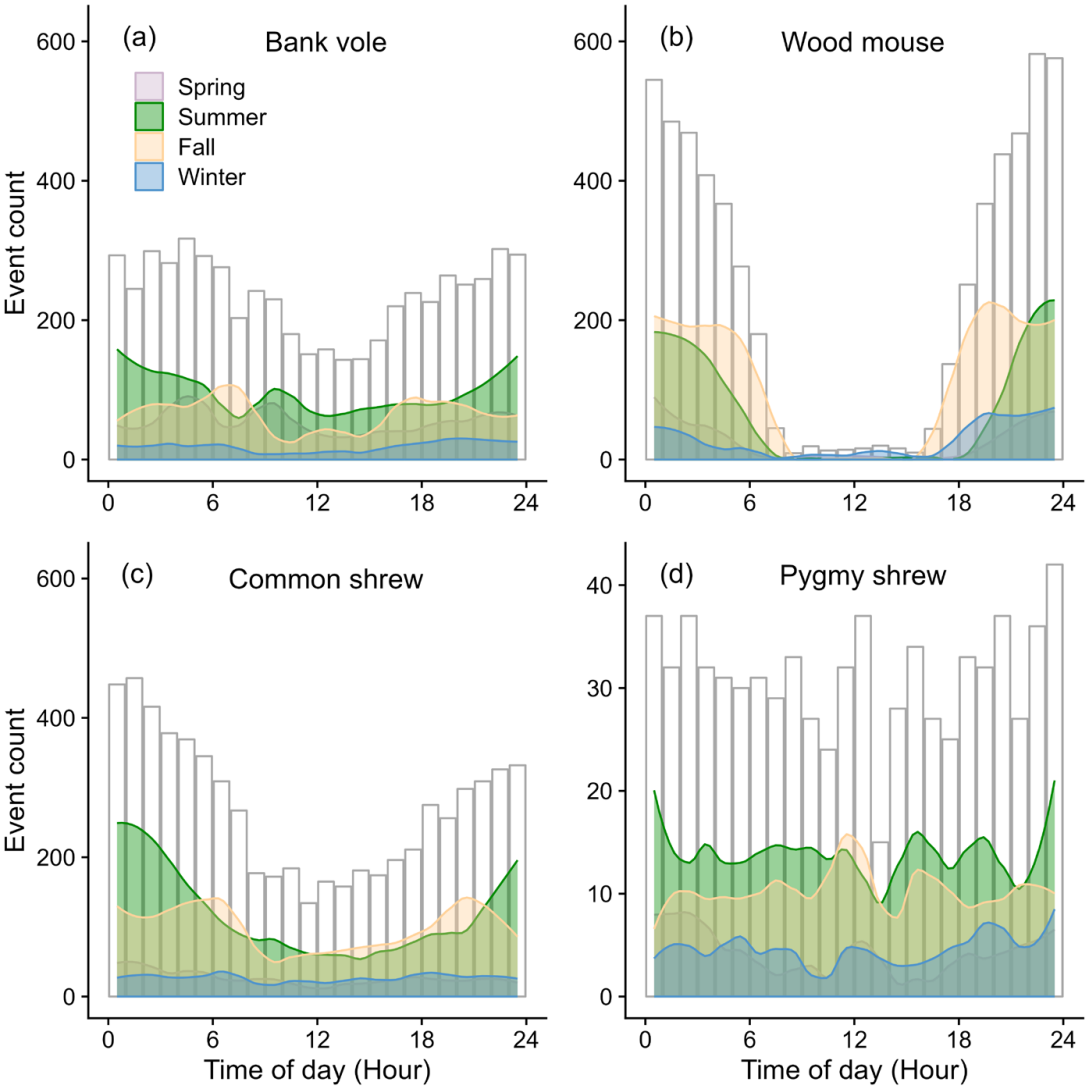
Number of events by time of day (hour) and season of (a) bank vole, (b) wood mouse, (c) common shrew, and (d) pygmy shrew recorded with camera traps in South‐East Norway (2018–2020). Daily camera trap events were smoothed using the loess method (*f* = 0.25). White bars denote event counts independently of season. Note the different *y*‐axis for the pygmy shrew.

**FIGURE 4:**
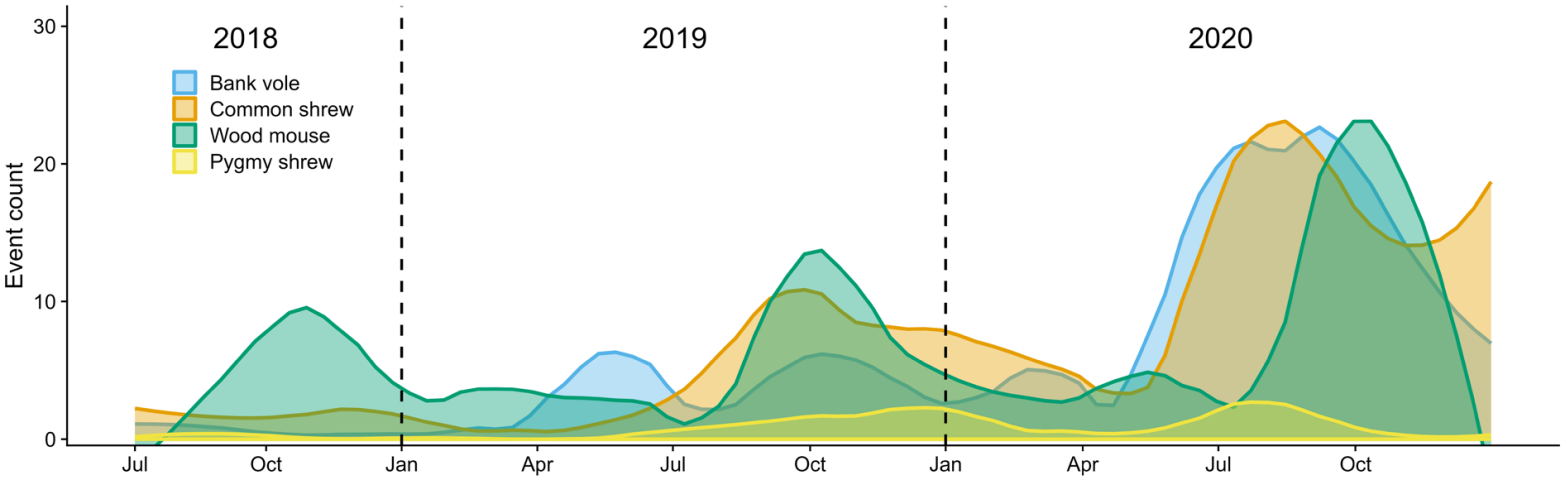
The number of daily events of bank vole, wood mouse, common shrew, and pygmy shrew recorded with camera traps in South‐East Norway (July 2018–December 2020). Daily camera trap events were smoothed using the loess method (*f* = 0.20).

Statistical analyses were conducted in R version 4.3.1 (R Core Team 2023). We restricted all statistical analyses to the four most frequently recorded small mammal species (bank vole, wood mouse common shrew, and pygmy shrew; Figure 2). Raw camera trap data were described by (1) summing up the hourly number of events for each species and for each season (Figure 3) and by (2) summing up the daily number of events over the entire study period in which each species was recorded (Figure 4). We defined winter as January–February, spring as March–June, summer as July–September, and fall as October–December. These seasons were set to fit the annual seasonality in this area close to the coastline and at low elevation, with snow cover mainly in January–February, melting, onset of plant growth, and deciduous trees getting leaves in spring, the full summer season in July–September, and frost and onset of plant senescence from October to December.

To statistically quantify the propensity of each species to select different diel periods, we fitted diel niche models in the package Diel.niche version 0.0.1.0 (Gerber et al. 2024). First, we fitted season‐specific diel niche models to test the support of traditional diel niches (cathemeral, crepuscular, diurnal, or nocturnal) based on the estimated probability to select each diel period (daytime, nighttime, and twilight) in summer and in winter. We defined categorization of crepuscular, diurnal, and nocturnal phenotypes (i.e., twilight‐, day‐, and night‐active, respectively) as having at least 0.60 probability of occurrence, respectively, or otherwise as cathemeral (irregular activity evenly distributed through the day). We fitted the diel niche models with number of chains = 5, Markov chain Monte Carlo (MCMC) iterations = 10,000, and burn‐in MCMC iterations = 3000. The diel period for each camera trap event was extracted at the study area centroid (latitude‐longitude 10.69, 59.56) using the package suncalc version 0.5.1 (Thieurmel et al. 2019). Second, we fitted species‐specific categorical regression models with day‐of‐year‐specific diel selection probabilities as a function of ordinal day (day‐of‐the‐year) to test whether the diel period selection of each species changed throughout the year.

To investigate if the number of captured individuals in ordinary traps were correlated with the number of camera trap occurrences of rodents (bank voles and wood mice) and shrews (common shrews and pygmy shrews), we fitted generalized linear models for Poisson distributed data with site‐specific number of trap‐captured individuals as a function of site‐specific camera trap occurrences and trap type. Site‐specific camera trap occurrences were summarized per species in the 30 days prior to onset of ordinary trapping. Trap type was included as a factor variable to account for possible variability in trappability in live cage traps versus lethal snap traps. Lastly, we calculated the McFadden's pseudo R‐squared for each model (McFadden 1974).

To test whether temperature and snow cover influenced small mammal occurrence in winter, we used negative binomial generalized linear mixed model fitted jointly for all four species using the package glmmTMB version 1.8.1 (Brooks et al. 2017). We used snow water equivalent (SWE) as our proxy variable for snow cover. The SWE is a variable that is more readily available and less influenced by snow drifting compared to snow depth, which is often a problem when measuring snow at a single location. In this case, the correlation between the two was 0.98 (*p* < 0.001), and hence provide the same information about environmental conditions.

We first fitted a global model that included species as a factor variable, daily mean temperature and daily SWE as continuous variables, and their respective 2‐ and 3‐way interactions as predictor variables, year as categorical random term, and daily event counts as response. Model selection was done using backward selection procedure with Akaike Information Criterion (AIC) using the package MuMIn version 1.47.1 (Barton 2022) for all seasonal models. We retained only the most parsimonious model within ΔAIC < 2.0 (Burnham and Anderson 1998). The model residuals were inspected to assess goodness‐of‐fit using the package DHARMa version 0.4.6 (Hartig 2022).

## 3 Results

### 3.1 Small Mammal Detections

The camera traps were triggered in total 70,426 times, on average 5869 times per camera (range 2013—12,405), resulting in a total of 211,288 pictures. A total of 24% of the images had no animal present. Only 0.7% of all pictures with animals were difficult to classify to species and were discarded. The 12 camera traps recorded 17,437 events and a total of 8 different mammal species (Table 1). The most recorded small mammal species was the common shrew, followed by bank vole, wood mouse, and pygmy shrew (Table 1). Other small‐ and medium‐sized mammals recorded included field voles, red squirrel, water shrew ( *Neomys fodiens* ), and common weasel ( *Mustela nivalis* ). Most observations were of one individual animal, while 283 observations were of two individuals of the same species in the same frame. Site‐specific observations are shown in the Supporting Information (Table S1).

**TABLE 1:** The number of camera trap events of recorded small‐ to medium‐sized mammals in South‐East Norway (2018–2020) by species, year, and season.

Species	2018		2019				2020				Total
	Sum	Fall	Win	Spr	Sum	Fall	Win	Spr	Sum	Fall	
Common shrew	158	180	67	145	683	834	572	516	1996	834	5985
Wood mouse	200	670	372	236	526	852	311	379	972	852	5370
Bank vole	89	28	74	454	312	453	366	888	1914	453	5031
Pygmy shrew	33	5	6	14	104	187	97	75	193	187	901
Field vole	1	0	0	0	0	2	30	28	21	2	84
Red squirrel	4	0	0	0	2	13	12	2	4	13	50
Water shrew	0	0	0	1	0	4	0	0	1	4	10
Common weasel	1	0	0	0	0	0	0	0	5	0	6

Abbreviations: spr, spring; sum, summer; win, winter.

### 3.2 Diel Period Selection

The bank vole was recorded at all hours of the day in all seasons (Figure 3a). The wood mouse was predominantly recorded between 6 pm and 6 am in all seasons (Figure 3b). The common shrew was recorded at all hours of the day in all seasons, albeit more often at night than during the day in summer and fall (Figure 3c). Lastly, the pygmy shrew was recorded at all hours of the day in all seasons but less frequently overall compared to the former species (Figure 3d).

The season‐specific diel niche models categorized the wood mouse as nocturnal in both seasons, the common shrew as cathemeral in both seasons, while both the bank vole and pygmy shrew were cathemeral in summer and nocturnal in winter (Figure S1). The probability of nocturnality varied between species and seasons. The probability of nocturnality was lower in summer compared to winter for bank voles and pygmy shrews, while it was stably over 0.8 throughout the year for wood mice and stably close to 0.5 for common shrews (Figure S2).

### 3.3 Annual and Seasonal Occurrence

The number of small mammal events was highest in 2020 (*n* = 10,727), followed by 2019 (5341) and 2018 (1369). The bank vole was recorded 117 times in 2018 with no clear seasonal peaks, followed by 1293 events in 2019 with small peaks in May and in September–October, and 4270 events in 2020 with a smaller peak in March and a larger peak in May through October (Figure 4). The wood mouse was recorded 870 times in 2018 with a seasonal peak in October, followed by 1986 events in 2019 with a peak in September–October, and 2890 events in 2020 with a smaller peak in May and a larger peak in September–October (Figure 4). The common shrew was recorded 338 times in 2018 with no clear seasonal peaks, followed by 1729 events in 2019 with a peak in September, and 4459 events in 2020 with a larger peak in July (Figure 4). The pygmy shrew was recorded 38 times in 2018 with no clear seasonal peaks, followed by 311 events in 2019 with most events in July through December, and 399 events in 2020 with a smaller peak in July–August (Figure 4).

### 3.4 Climatic Effects on Winter Occurrence

**FIGURE 5:**
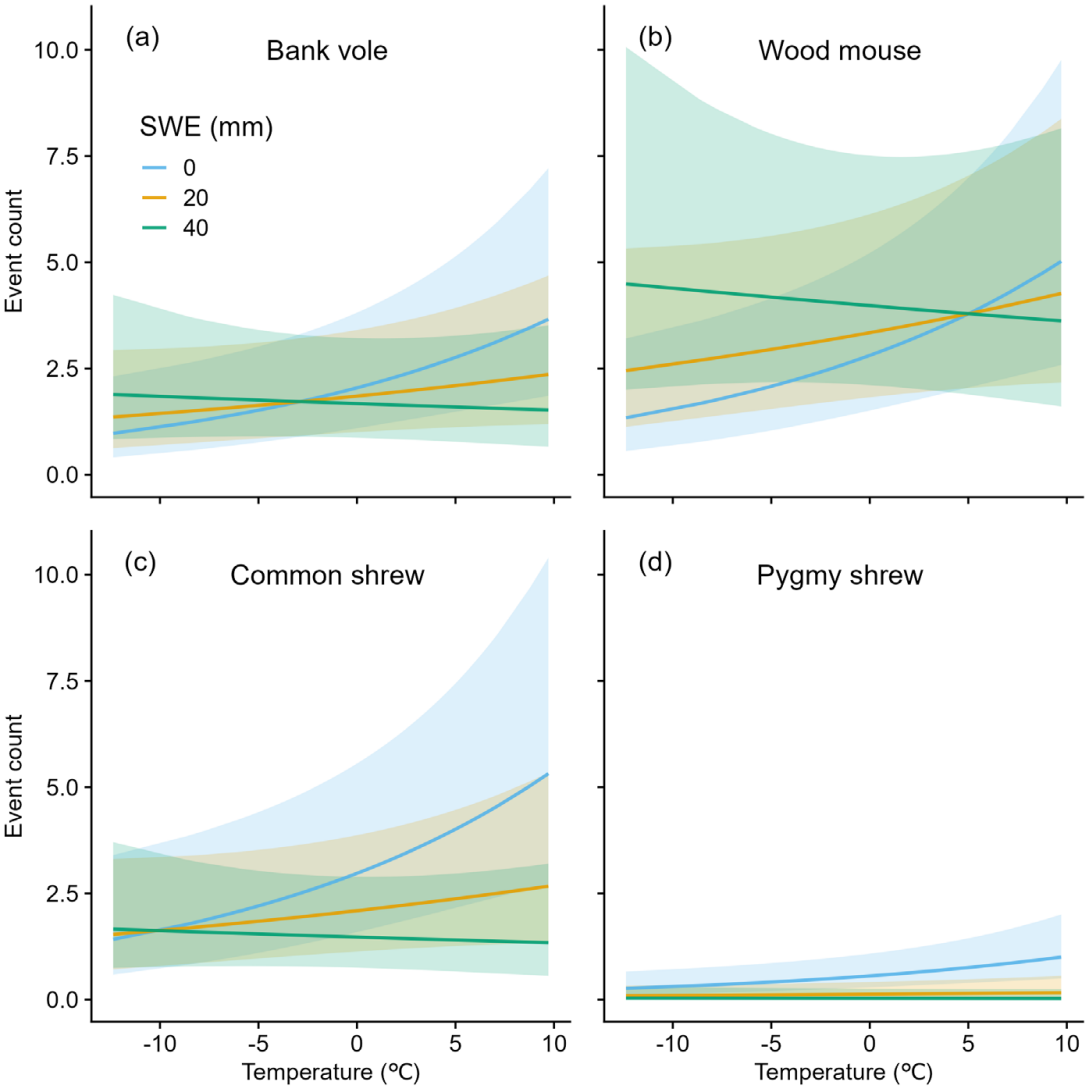
The model predictions of winter‐specific (January–March) camera trap events of (a) bank vole, (b) wood mouse, (c) common shrew, (d) and pygmy shrew in South‐East Norway (2019 and 2020), as a function of mean daily temperature depending on snow water equivalent. Shaded polygons denote respective 95% confidence intervals.

In the winter‐specific models on camera trap occurrence of bank voles, wood mice, common shrews, and pygmy shrews, occurrence was influenced by species, daily mean temperature, daily SWE, and the interaction between “temperature × SWE” and “species × SWE” (Table S2). The effect of temperature was positive for all species, but dependent on SWE, whereby it increased more when snow cover was poor (Figure 5). The effect of snow water equivalent was positive only for wood mice when temperatures were low, whereas it was negative at all temperatures for bank voles, common shrews, and pygmy shrews (Table 2). Sub‐zero temperatures and a consistent winter snow cover were common in the winter (January March) of 2019, but not in the winter of 2020 (Figure S3).

**TABLE 2:** Estimates of parameters from negative binomial generalized linear models on winter‐specific (January–March) occurrence of small mammals recorded with tunnel‐based camera traps in South‐East Norway (2019 and 2020) as a function of species (sp), temperature (Temp) and snow water equivalent (SWE). The intercept baseline corresponds to species = bank vole.

Parameter	Estimate	Std. error	*z*	*p*
Intercept	0.716	0.318	2.251	0.024
spCommon shrew	0.373	0.136	2.739	0.006
spPygmy shrew	−1.299	0.170	−7.636	< 0.001
spWood mouse	0.317	0.143	2.209	0.027
SWE	−0.005	0.005	−1.056	0.291
Temp	0.060	0.021	2.890	0.004
SWE: Common shrew	−0.013	0.006	−2.106	0.035
SWE: Pygmy shrew	−0.069	0.028	−2.471	0.013
SWE: Wood mouse	0.014	0.005	2.606	0.009
SWE: Temp	−0.002	0.001	−2.750	0.006

### 3.5 Camera and Ordinary Trapping Correlations

In the ordinary traps, we captured 111 bank voles, 38 common shrews, 29 wood mice, and 12 pygmy shrews (Table S3). Across both rodents and shrews, the number of captured small mammals corresponded well with the recorded occurrence by camera traps (*p* < 0.001, pseudo *R* ^2^ = 0.10, *z* = 10.8, Figure S4a) and tended to be positively influenced by trap type (*p* = 0.051; Table S4). For rodents (bank voles and wood mice) only, the number of individuals captured with ordinary traps corresponded well with the recorded occurrence by camera traps (*p* < 0.001, pseudo *R* ^2^ = 0.13, *z* = 8.4, Figure S4b) and was positively influenced by snap traps (*p* < 0.001, *z* = 3.8; Table S4). For shrews (common shrews and pygmy shrews) only, the number of captured individuals corresponded well with the recorded occurrence by camera traps (*p* < 0.001, pseudo *R* ^2^ = 0.09, *z* = 4.1, Figure S4c) and was negatively influenced by snap traps (*p* = 0.004, *z* = −3.0; Table S4).

## 4 Discussion

Most data series on small mammal occurrences are based on population indices often recorded through only a few trapping sessions per year (Krebs 1996, 2019). The possibility to obtain continuous data on small mammals enables quantitative analyses of small mammal occurrence at unprecedented temporal resolutions (Kleiven et al. 2023). The camera traps deployed in the present study provided data enabling identification of diel niches between species and seasons, cyclic phases, and seasonal patterns over years, and to relate small mammal occurrence to climatic variables.

Recently, methods to objectively categorize different diel niches have surfaced (Gerber et al. 2024), and we here document how the level of synchrony among four species of small mammals varies depending on the season. In both summer and winter, the wood mouse was categorized as nocturnal, while the common shrew was cathemeral, as predicted (I). Cathemerality in shrews was expected, according to the relatively higher metabolism and thus higher foraging frequency in shrews compared to rodents (Gliwicz and Taylor 2002). Similarly, microtine rodents like voles typically exhibit cathemeral activity patterns (Curtis and Rasmussen 2006; Halle 2006), consistent with the observed summer cathemerality in bank voles. However, both pygmy shrews and bank voles were nocturnal in winter, contrary to our prediction (II) and previous studies on tundra voles ( *Microtus oeconomus* ) in northern Norway (Soininen et al. 2015). The observed winter nocturnality in bank voles and pygmy shrews may partly be due to poor snow cover in the study period. Without the subnivean space created by a persistent snow cover, small mammals active above the snow are highly visible and exposed to predators (Zimova et al. 2016; Penczykowski et al. 2017). However, a shift from cathemerality in summer to nocturnality in winter may also simply reflect increased night length in winter at the expense of daytime availability. Nevertheless, tunnel‐based camera traps combined with novel quantitative frameworks provide new insight into variation in season‐specific diel rhythms of different small mammals.

In addition to occurrence data at the time‐of‐day scale, the camera traps yielded long‐term trends in occurrence. Small mammal population cycles in Scandinavia are typically 3–4.5 years (Bjørnstad et al. 1997). Here, we document temporal synchrony of both bank voles, wood mice, and common shrews, from a low year in 2018, to an increase in 2019, and to a peak year in 2020. Occurrence of pygmy shrews developed similarly in the period, albeit at much lower amplitudes as expected. Annually, abundances of small mammals are typically at the lowest before onset of reproduction in early summer, and then are expected to increase with reproduction onset over the summer and reach a seasonal peak in fall (Crespin et al. 2002; Andreassen et al. 2021). We found that bank vole occurrence increased to a seasonal peak in early summer 2020 lasting through fall. In contrast, seasonal occurrence of wood mice was low between summer and early fall and only increased to a peak later in fall. Indeed, previous studies on wood mice suggest seasonal shifts in habitat selection from arable fields in the growing season to forests following the harvest period (Tew and Macdonald 1993; Fitzgibbon 1997; Tattersall et al. 2001), whereas bank voles and common shrews tend to use arable fields to a lesser extent in favor of forest habitat (Hansson 1985; Churchfield 1990; Fitzgibbon 1997). The different seasonality in occurrence peaks between species might thus reflect species‐specific seasonal shifts in space use (Chesson 2000). Hence, tunnel‐based camera traps enabled us to identify seasonal events in sympatric species of small mammals, that likely reflect a combination of activity and population dynamics through reproduction and mortality through the year, and highlight their taxonomic differences in occurrence through the year.

Seasonal shifts in abiotic conditions can strongly influence the activity of small mammals (Orrock et al. 2004; Penczykowski et al. 2017), through physiological effects, food availability, and predation risk (Churchfield 1982; Stokes et al. 2001; Orrock et al. 2004; Korslund and Steen 2006). As predicted (IV), there was a positive effect of temperature on winter occurrence, possibly due to physiological effects as higher‐than‐normal temperatures alleviate physiological stress associated with low winter temperatures (van der Vinne et al. 2014; Guiden and Orrock 2020). Moreover, the relationship with temperature was dependent on snow cover and there was no effect when snow cover was high. The subnivean space under the snow is thought to provide an isolating microhabitat that protects small mammals from extreme temperatures, prevent ice formation that limits food availability, and protects small mammals from avian predation (Ims and Fuglei 2005; Korslund and Steen 2006; Krebs 2010; Penczykowski et al. 2017). The snow cover‐dependent temperature effects might indicate that mild winter temperatures in our region may alleviate some of the challenges for small mammals imposed by warmer winters under climate warming, which often brings less frequent snow cover and reduced survival (Korslund and Steen 2006; Krebs 2010; Cornulier et al. 2013; Penczykowski et al. 2017). Compared specifically to periods with unstable temperatures and a shallow and compact snow cover, mild and completely snow‐free periods may promote ice formation that limits food availability (Ims and Fuglei 2005) and decrease predation through reduced contrast and thus visibility to predators on snow‐free ground, whereas they would generate a sharp contrast above shallow and compact snow and be highly visible to predators (Zimova et al. 2016). One the other hand, snow cover alone positively influenced wood mouse occurrence only. This might reflect very contrasting winters in the study area with a colder winter in 2019 and a mild and snow‐poor winter in 2020, and the relationship between small mammal occurrence and snow cover would possibly differ with a more persistent snow cover and over a longer study period. A longer time series with more environmental variation between winters could provide novel opportunities to explore these relationships in further detail.

The frequency of occurrence from camera traps correlated moderately with the capture counts from spatially coinciding ordinary trapping across all four species. Camera trap and ordinary small mammal trapping surveys have previously been deployed in parallel, and with similar correlations, in studies on gray‐sided voles ( *Myodes rufocanus* ) and tundra voles ( *Microtus oeconomus* ) in northern Europe and North America (Villette et al. 2016; Parsons et al. 2021; Kleiven et al. 2023) and deer mice ( *Peromyscus maniculatus* ) in North‐America (Villette et al. 2016). The lower correlation in shrews compared to rodents may reflect species‐specific biases in trapability in live traps, as the relative efficacy of live‐trapping methods may differ between taxa (Jung 2016). The correlations could also simply reflect data paucity due to a limited time series and that each live trapping session was only three consecutive days and thus prone to random variation in weather and animal activity. Nevertheless, camera trapping likely provides more robust monitoring less influenced by short‐term fluctuations, and may solve issues related to species‐specific trapability biases common to ordinary trapping surveys.

Camera trapping data offer novel opportunities to study patterns of occurrence and levels of synchrony at unprecedented temporal resolutions and provide novel insights into ecological niche partitioning and interspecific competition. We have focused on four species of small mammals with widespread distributions in northern and continental Europe (Deffontaine et al. 2005; Herman 2017), including two widespread species of shrew (Neves et al. 2022). Newly developed statistical tools allowed us to quantitatively categorize the type of diel niche (Gerber et al. 2024) and how the degree of nocturnality differed across seasons and species, which provides a new level of insight into the ecology of a species group of key importance for ecosystem dynamics.

## Author Contributions

**Lars K. Lindsø:** conceptualization (equal), data curation (equal), formal analysis (lead), investigation (lead), visualization (lead), writing – original draft (lead). **Inger Maren Rivrud:** formal analysis (supporting), investigation (supporting), methodology (supporting), supervision (supporting), writing – review and editing (equal). **Emilie A. König:** data curation (equal), writing – review and editing (equal). **Anders Herland:** data curation (equal), writing – review and editing (equal). **Atle Mysterud:** conceptualization (equal), data curation (equal), formal analysis (supporting), funding acquisition (lead), investigation (supporting), methodology (supporting), project administration (lead), supervision (lead), writing – review and editing (equal).

## Conflicts of Interest

The authors declare no conflicts of interest.

## Supporting information


Data S1.

## Data Availability

Data and scripts to reproduce results presented in this paper are available at Zenodo: https://doi.org/10.5281/zenodo.11354073.
