# Supplementary material for: Diel Niche of Sympatric Small Mammals Revealed by Year‐Round Camera Trapping
Source: Ecol Evol. 2025 Jun 12;15(6):e71590. doi: 10.1002/ece3.71590 (PMC12162949; doi:10.1002/ece3.71590)
Supplement: Supplementary file 1 — Data S1. [file ECE3-15-e71590-s001.pdf]

Supporting information

Diel niche of sympatric small mammals revealed by year-round camera trapping

Lars K. Lindsø, Inger Maren Rivrud, Emilie A. König, Anders Herland, and Atle Mysterud

Table S1: The number of camera trap events of recorded small- to medium-sized mammals in South-East Norway (2018-2020) by species and camera trap site.

		**Camera trap site**											
**Species**	**Sum**	**1**	**2**	**3**	**4**	**5**	**6**	**7**	**8**	**9**	**10**	**11**	**12**
Common shrew	7554	1200	1220	484	460	423	653	674	675	371	95	1198	101
Bank vole	5965	274	665	495	144	336	1364	490	276	993	168	577	183
Wood mouse	5876	187	1314	1064	411	281	202	334	597	1131	178	77	100
Pygmy shrew	918	9	84	1	45	48	7	137	52	11	28	361	135
Field vole	106	0	0	0	0	0	0	30	2	35	1	33	5
Red squirrel	44	0	23	0	0	7	0	5	8	1	0	0	0
Water shrew	24	0	3	1	15	0	0	0	0	1	1	3	0
Common weasel	10	3	2	1	3	0	1	0	0	0	0	0	0

Table S2: Results of model selection of number of camera trap events of small mammals in winter (January–March) in South-East Norway (2018–2020), using generalized linear models for negative binomially distributed data, with species (**sp**) as a factor variable, snow water equivalent (**swe**) and temperature (**tmp**) as numeric parameters, and year as a categorical random intercept. The table shows only the top ten models. Included model parameters and interactions are denoted “+” and the model used for inference is highlighted in grey.

Int	sp	swe	tmp	sp:swe	sp:tmp	swe:tmp	sp:swe:tmp	df	AICc	ΔAIC	wgt
0.824	+	+	+	+	+	+		15	2512.1	0.0	0.47
**0.716**	**+**	**+**	**+**	**+**		**+**		**12**	**2513**	**0.6**	**0.4**
0.862	+	+	+	+	+	+	+	18	2514.8	2.7	0.13
0.771	+	+	+	+				11	2518.3	6.2	0.02
0.860	+	+		+				10	2519.1	7.0	0.01
0.883	+	+	+	+	+			14	2520.4	8.2	0.01
0.627	+	+	+			+		9	2545.9	33.7	0
0.713	+	+	+		+	+		12	2546.2	34.1	0
0.764	+		+		+			10	2548.6	36.4	0
0.707	+		+					7	2549.8	37.7	0

Table S3: The number of bank voles. common shrews, wood mice, and pygmy shrews captured with traps in proximity to camera traps in spring and fall between 2018 and 2020 in Son, Viken county, Norway.

		**2018**	**2019**		**2020**	
**Species**	**Sum**	**Fall**	**Spring**	**Fall**	**Spring**	**Fall**
Bank vole	111	13	4	7	8	79
Common shrew	38	9	0	11	1	17
Wood mouse	29	7	5	4	1	12
Pygmy shrew	12	2	0	2	1	7

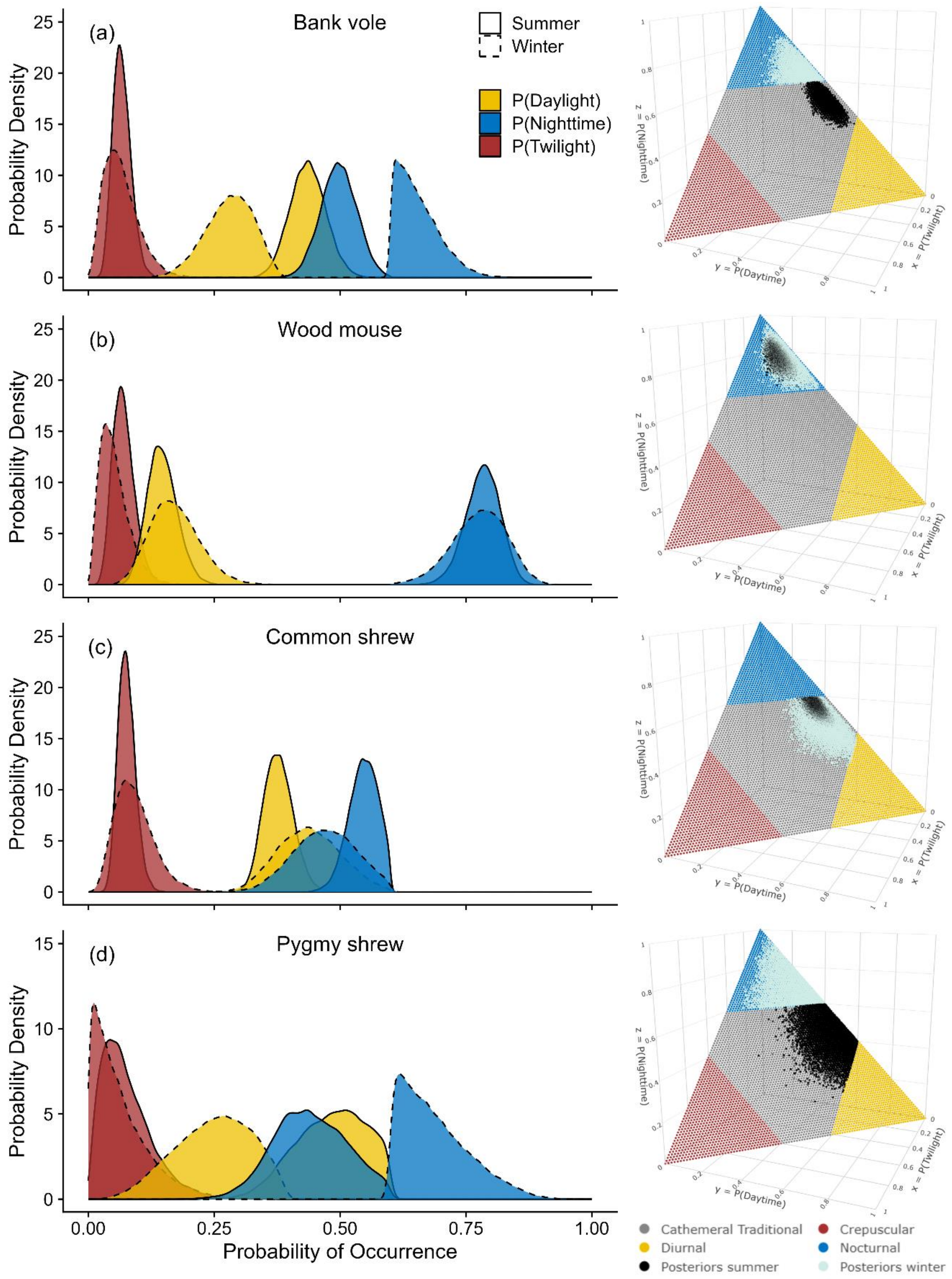

Figure S1: The estimated probability to select diel periods (daylight, nighttime, and twilight; left-hand panels) and traditional diel niche (right-hand panels) in **a** bank voles, **b** wood mice, **c** common shrews, and **d** pygmy shrews in summer and winter recorded with camera traps in South-East Norway (2018-2020). Overlayed dots on the right-hand panels denote model-fitted posterior samples for each season.

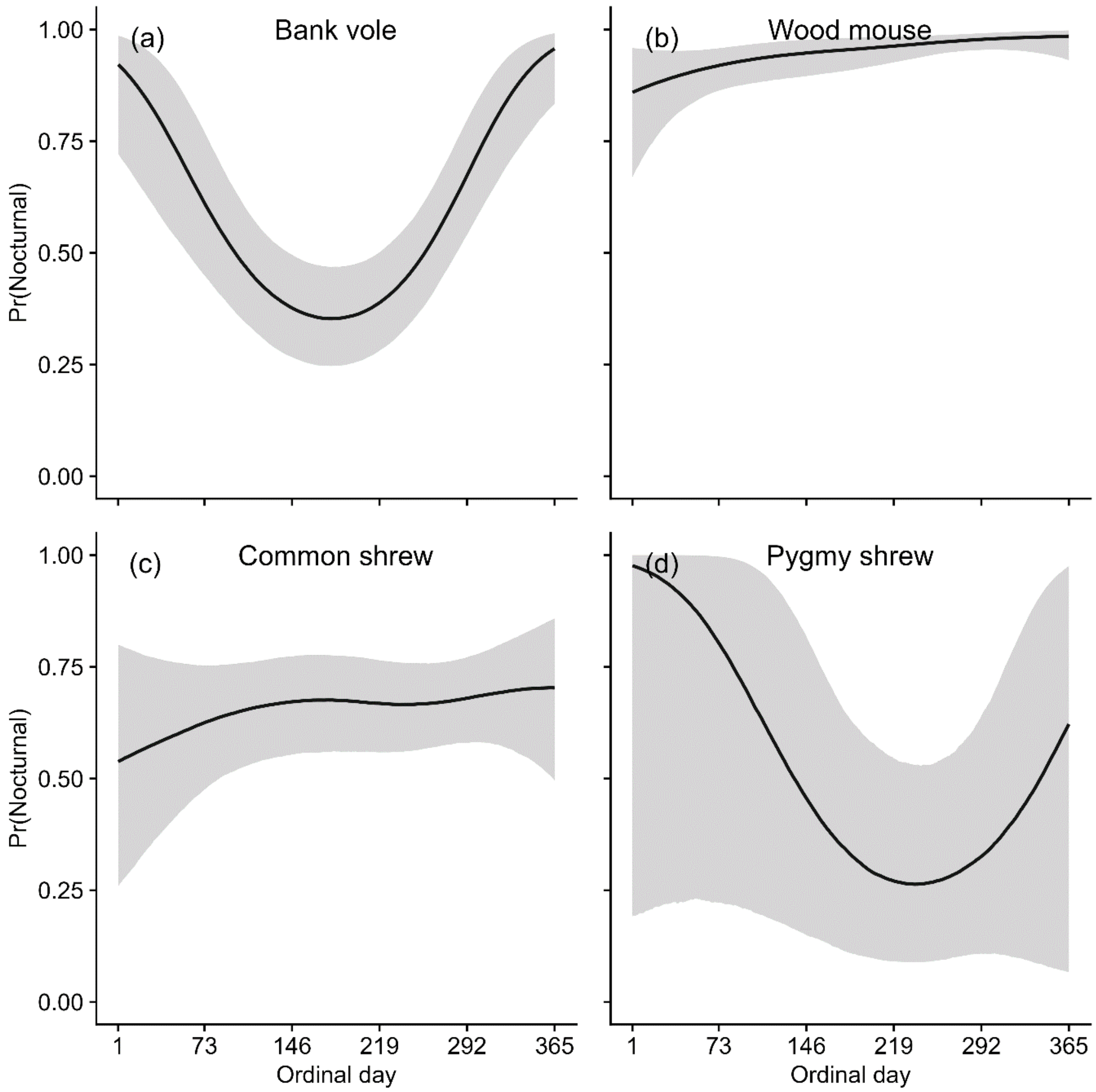

Figure S2: The estimated probability of nocturnality Pr(Nocturnal) as a function of day-of-the-year (Ordinal day) in bank vole, wood mouse, common shrew, and pygmy shrew recorded with camera traps in South-East Norway (July 2018 - December 2020). Shaded polygons denote respective 95% confidence intervals.

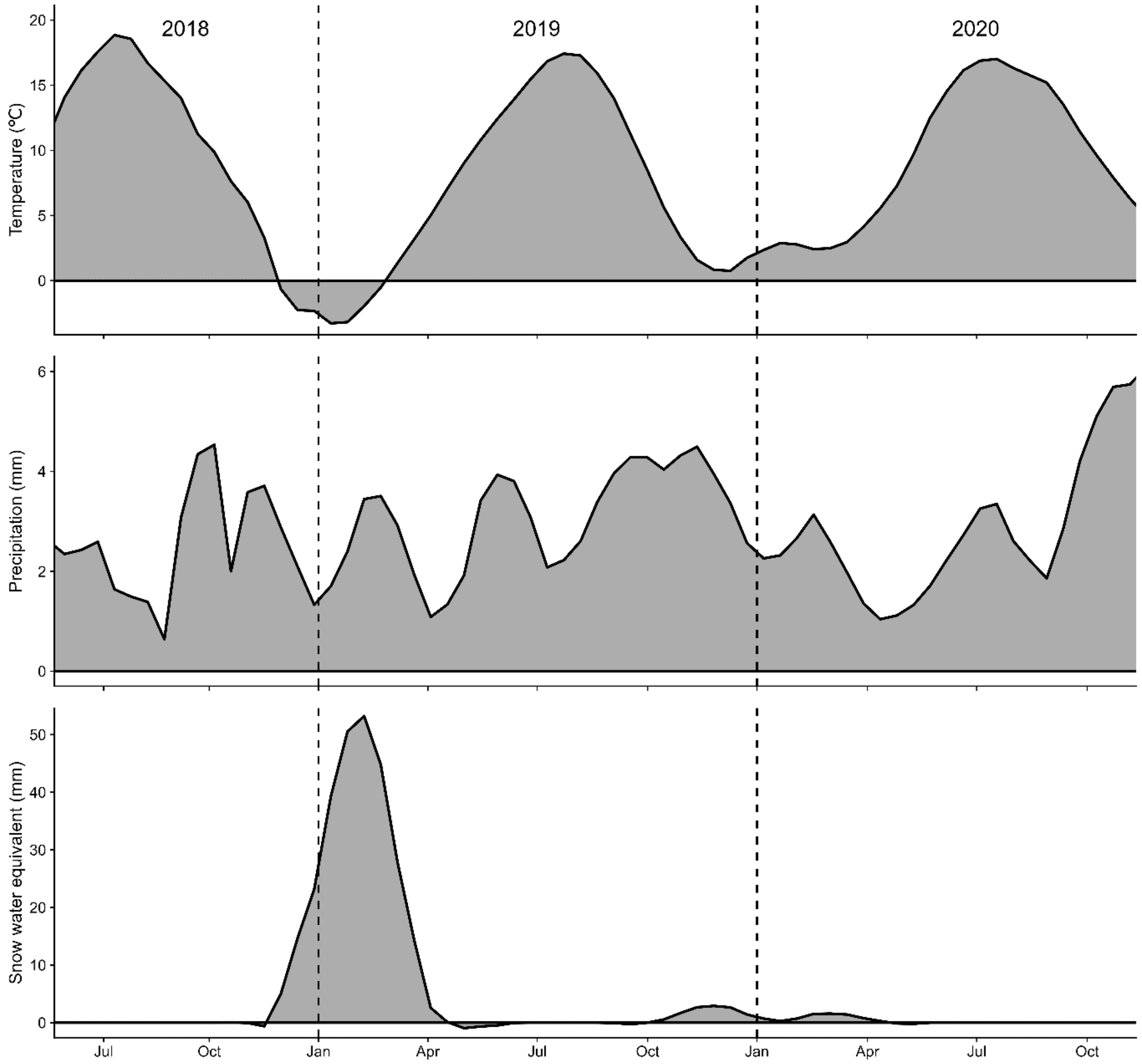

Figure S3: The aggregated daily mean temperature (°C), precipitation (mm), and snow water equivalent (mm) in South-East Norway (July 2018 - December 2020). Climate variables were smoothed using the loess method (f = 0.15).

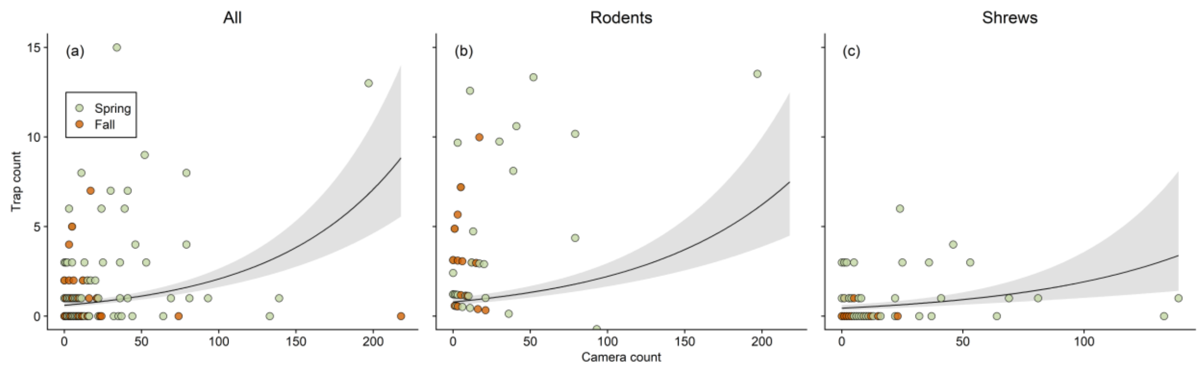

Figure S4: The model predictions of number of captured **(a)** rodents (bank voles and wood mice), **(b)** shrews (common shrews and pygmy shrews), and **(c)** both rodents and shrews as a function of camera trap events in South-East Norway (2018-2020). Shaded polygons denote respective 95% confidence intervals and points denote season-specific raw data. Predictions are estimated with trap type = live cage trap.

Table S4: Estimates of parameters from generalized linear models for poisson distributed data on number of captured small mammals with ordinary traps at 12 sites in South-East Norway (2018-2020) as a function of camera trap occurrence and trap type. The baseline trap type was live cage trap.

**Parameter**	**Estimate**	**Std. error**	***z***	***P***
All				
Intercept	-0.496	0.099	-5.012	<0.001
Camera trap occurrence	0.012	0.001	10.790	<0.001
Trap type = snap	0.274	0.141	1.951	0.051
Rodents				
Intercept	-0.234	0.123	-1.904	0.057
Camera trap occurrence	0.010	0.001	8.434	<0.001
Trap type = snap	0.618	0.162	3.819	<0.001
Shrews				
Intercept	-0.808	0.170	-4.743	<0.001
Camera trap occurrence	0.015	0.004	4.149	<0.001
Trap type = snap	-1.128	0.387	-2.914	0.004
